# Divergent antibody recognition profiles are generated by protective mRNA vaccines against Marburg and Ravn viruses

**DOI:** 10.21203/rs.3.rs-4087897/v1

**Published:** 2024-03-26

**Authors:** Alexander Bukreyev, Michelle Meyer, Bronwyn Gunn, Colette Pietzsch, Chandru Subramani, Erica Saphire, James Crowe, Galit Alter, Sunny Himansu, Andrea Carfi

**Affiliations:** University of Texas Medical Branch; University of Texas Medical Branch; Washington State University; University of Texas Medical Branch at Galveston; University of Texas Medical Branch at Galveston; La Jolla Institute for Immunology; Vanderbilt University Medical Center; Moderna/Ragon Institute; Moderna; Moderna, Inc.

## Abstract

The first-ever recent Marburg virus (MARV) outbreak in Ghana, West Africa and Equatorial Guinea has refocused efforts towards the development of therapeutics since no vaccine or treatment has been approved. mRNA vaccines were proven successful in a pandemic-response to severe acute respiratory syndrome coronavirus-2, making it an appealing vaccine platform to target highly pathogenic emerging viruses. Here, 1-methyl-pseudouridine-modified mRNA vaccines formulated in lipid nanoparticles (LNP) were developed against MARV and the closely-related Ravn virus (RAVV), which were based on sequences of the glycoproteins (GP) of the two viruses. Vaccination of guinea pigs with both vaccines elicited robust binding and neutralizing antibodies and conferred complete protection against virus replication, disease and death. The study characterized antibody responses to identify disparities in the binding and functional profiles between the two viruses and regions in GP that are broadly reactive. For the first time, the glycan cap is highlighted as an immunoreactive site for marburgviruses, inducing both binding and neutralizing antibody responses that are dependent on the virus. Profiling the antibody responses against the two viruses provided an insight into how antigenic differences may affect the response towards conserved GP regions which would otherwise be predicted to be cross-reactive and has implications for the future design of broadly protective vaccines. The results support the use of mRNA-LNPs against pathogens of high consequence.

## INTRODUCTION

Marburg virus (MARV) and Ravn virus (RAVV), which belong to genus *Marburgvirus*, along with several other viruses, including Ebola virus (EBOV) and Sudan virus (SUDV), are members of the *Filoviridae* family that cause lethal hemorrhagic fever in humans. There are currently no licensed vaccines against MARV, but several vaccine constructs have demonstrated protective efficacy in non-human primates (NHPs) (1–6). Given the recent concurrent MARV outbreaks in two non-endemic countries of Africa, Equatorial Guinea (7) and the United Republic of Tanzania (8), and a 24 to 88% case fatality rate (9), the perceived risk of MARV spreading similarly to the unprecedented 2013 to 2016 West-African EBOV outbreak is justified. Advances with several vaccine platforms relying on virus glycoprotein (GP) antigens to induce a protective immune response have been achieved, several of which have shown promise in Phase I clinical trials or are on the cusp of entering trials. However, these candidates need to be tested during an outbreak setting. Virus-vectored vaccines have been tailored against MARV, some with proven efficacy in ring vaccination trials conducted during the 2013 to 2016 West African outbreak of a close virus relative, EBOV. Two vesicular stomatitis virus (VSV)-derived vaccines against MARV strain Angola and strain Musoke, similar to the fully-licensed *Ebola Zaire Vaccine, Live* (tradename: ERVEBO; Merck Sharp & Dohme) against EBOV, await clinical trials (10, 11). Efforts are underway to reduce the vaccine-associated side-effects by implementing a further attenuated version of the platform (12).

Heterologous prime-boost combinations of replication-incompetent multivalent adenovirus type 26 (Ad26)- and Ad35-MARV based vaccines, engineered similarly to the Ad26 component of the approved heterologous prime-boost EBOV vaccine developed by Johnson & Johnson (J&J) Janssen: *ebola vaccine (Ad26.ZEBOV-GP [recombinant])* (Zabdeno) with *Ebola vaccine (MVA-BN-Filo [recombinant])* (Mvabea), provided NHPs with a 75–100% chance of survival against MARV infection (2). Phase II clinical trials of the chimpanzee adenovirus 3 version of recombinant adenoviruses vaccines, which mitigates vector immunity, are underway in Africa after the vaccine successfully demonstrated safe and long-term antibody responses in adults in the USA (13) and rapid and durable efficacy in NHPs (4).

The Mvabea constituent of the Janssen vaccine is a modified vaccinia Ankara viral vector expressing antigens from four different filoviruses including the GP from MARV strain Musoke (93% amino acid homology to Angola GP), but its efficacy against MARV was never fully assessed (14, 15). DNA-based vaccines yielded limited seroconversion in Phase I clinical trials despite multiple boosters (16). Virus-like or inactivated virus particles require multiple, adjuvanted doses to confer NHPs with protection against MARV and heterologous RAVV (1, 3)

We have previously shown that a two-dose vaccination regimen of a modified mRNA encoding EBOV GP formulated with a lipid encapsulation could effectively protect guinea pigs against EBOV (17). Our results provided evidence that the mRNA platform could be a formidable vaccine against pathogens of high consequence. The need remains for vaccine platforms that target closely related members of the *Filoviridae* family, given that the current licensed vaccines are virus vector-based and shown to be efficacious against EBOV only, in clinical settings.

Eliminating virus vector backbones as the delivery vehicle for encoded antigen has advantages. Vector-less delivery provides the capacity to design a more targeted response without complications from vector-generated adverse effects. Issues of preexisting immunity toward the vector delivery vehicle are also circumvented allowing for repeat dosing and a reimagined vaccine platform against a spectrum of pathogens.

Ever since protein expression from exogenously introduced naked mRNA was demonstrated in mouse muscle (18), the development of mRNA as vaccines against infectious diseases has gained steady momentum. mRNA vaccines were modified to bypass the body’s immune defenses and encapsulated by lipid nanoparticles to facilitate delivery. While mRNA designed to target viruses including influenza virus, respiratory syncytial virus and Zika virus advanced through Phase I and II trials (19–21), it will be years before they are clinically accessible. The full potential of mRNA vaccines was realized at the onset of the COVID-19 pandemic, which propelled regulatory approval and widespread administration of what at the time was still considered a novel platform (22).

In this study, we expand on assessing the effectiveness of mRNA vaccines against two closely-related marburgviruses by testing the efficacy of a two-dose regimen of MARV-specific and RAVV-specific vaccines in guinea pigs. Cross-protection against both MARV and RAVV is of concern given that the GP of MARV (strain Angola) and RAVV (strain Ravn) are 22% divergent at the amino acid level and 10% divergent when the mucin-like domain (MLD) is excluded (23). Therefore, mRNA vaccines were designed to target the GP of MARV or the genetically distinct RAVV and tested against the respective viruses. In addition to survival analysis, we characterize the functional properties of the antibody response and map targeted antigenic sites to reveal the commonalities and uniqueness between the MARV and RAVV mRNA vaccine-induced profiles. Our results further support the advancement of the mRNA vaccine platform development against highly lethal viruses.

## RESULTS

### mRNA design, generation, and vaccination schedule

**Fig 1.**
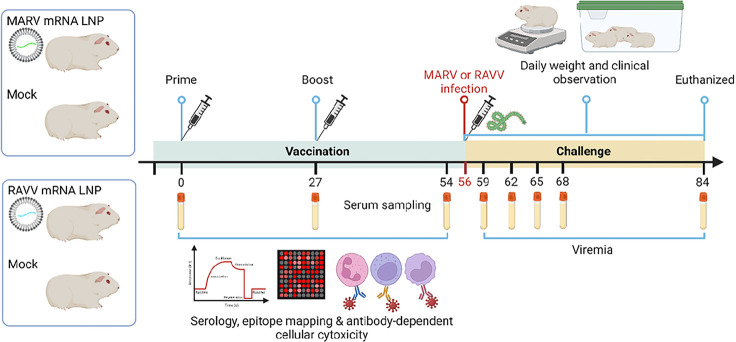
Schematic of study design. Guinea pigs were vaccinated (n=5 per group) via the IM route on day 0 and boosted on day 27 with MARV mRNA (green) or RAW mRNA (blue). Control groups were mock vaccinated. Animals were challenged with 1,000 PFU of guinea pig-adapted MARV or RAW by the IP route at day 56. All animals were monitored for changes in weight, clinical scores, viremia and survival over 28 days. Serum was collected from each animal at 3-day intervals over the course of infection and measured for infectious virus.

mRNA vaccines encoding the GP of MARV or RAVV were synthesized *in vitro* from linearized DNA templates of the mRNA by T7 polymerase-mediated transcription in which the UTP was substituted with 1-methylpseudo-UTP. mRNA constructs were then encapsulated in LNP formulations for subsequent delivery *in vivo* as previously (24). Two groups of Hartley guinea pigs (n = 5) were prime vaccinated via the intramuscular route on day 0 and boosted on day 27 with mRNA-LNP vaccine against MARV or against RAVV (Fig. 1). Two control groups of guinea pigs (n = 5) were mock vaccinated with PBS. Over the vaccination phase of the study, serum was collected, and the antibody binding and functional profiles were characterized.

### Both mRNA vaccines generate autologous and asymmetric heterologous virus-neutralizing antibody responses

**Fig. 2.**
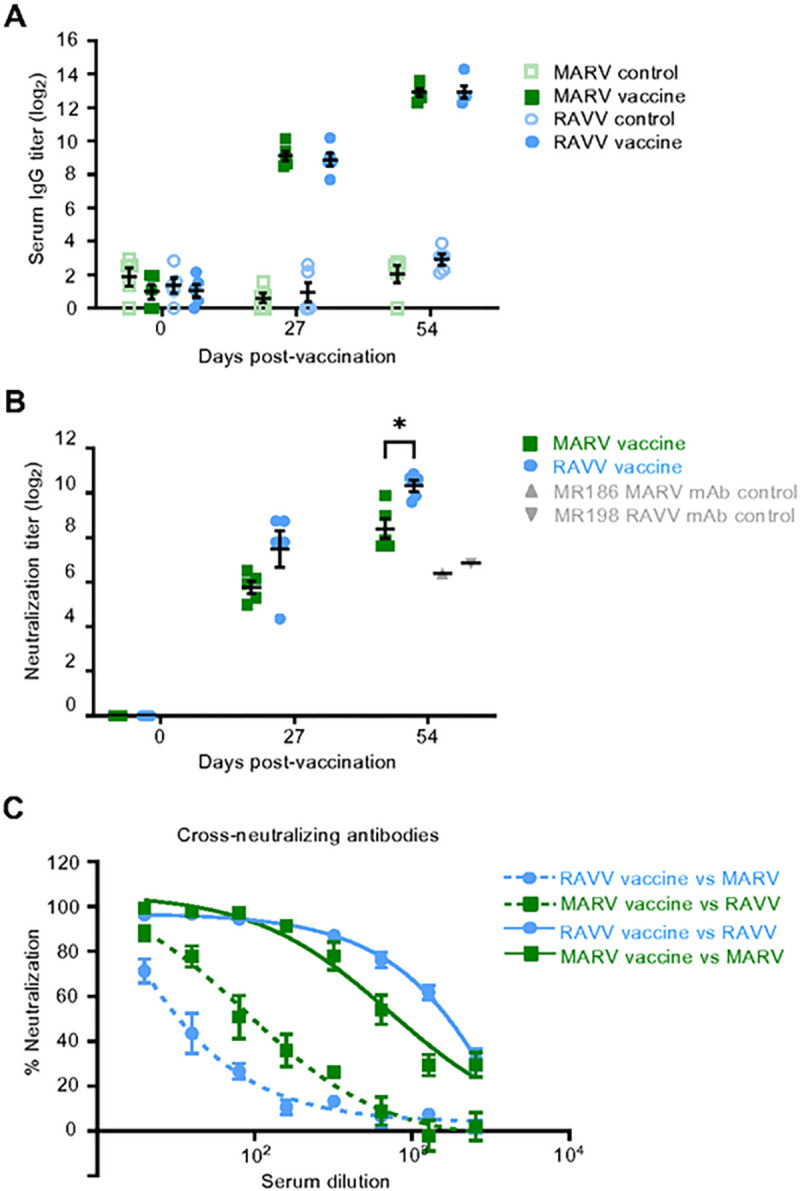
Serum antibody responses in vaccinated guinea pigs. **(A)** Total GP-specific IgG in serum, **(B)** virus-neutralizing antibody titers against homologous or **(C)** heterologous viruses targeted by the vaccines were measured in vaccinated groups prior to MARV (green) or RAW (blue) inoculation using ELISA or plaque reduction neutralization assays, respectively. Bars denote group means ± SEM. **(B)** Antibody titers required to achieve 60% virus neutralization. **(C)** Serum virus-neutralizing antibody dilution curves against homologous (solid lines) or heterologous viruses (dashed lines). Significance between vaccine recipients measured by repeated measures two-way ANOVA with Sidak’s correction for multiple comparisons **(A, B).** *P ≤ 0.05**.**

Following each vaccination dose, we monitored virus-specific antibody responses in serum. High anti-MARV or -RAVV IgG titers were detected by ELISA 27 days after the prime dose of the respective mRNA vaccine (Fig. 2A), which was further elevated by the booster dose. MARV- and RAVV-specific IgG titers were comparable after both the prime and boost vaccinations. Both vaccines also induced neutralizing antibodies against the respective viruses, which somewhat mirrored the IgG titers in that titers after the prime vaccination were further elevated by the booster (Fig. 2B).

Given that MARV and RAVV are genetically distinct but share 78% GP sequence identity at the amino acid level, we determined the ability of the serum collected after the boost vaccination to neutralize heterologous virus (Fig. 2C). The MARV vaccine autologous virus-neutralizing antibody titer was lower compared to the autologous virus-neutralizing titer generated by the RAVV vaccine (Fig. 2B). However, the MARV vaccine induced a higher cross-neutralizing titers against RAVV (reciprocal neutralizing titer 50 [NT_50_] of 89.0) compared to the RAVV vaccine neutralizing titers against MARV (NT_50_ of 8.2) (Fig. 2C).

These data demonstrate that MARV and RAVV mRNA vaccines elicit comparable binding antibody responses after prime and boost doses against their respective viruses. The RAVV vaccine yielded higher autologous neutralizing antibodies than MARV, but the MARV response appeared to be more cross-neutralizing.

### The mRNA vaccines differ in their response towards the proteolytically cleaved form of GP

**Fig. 3.**
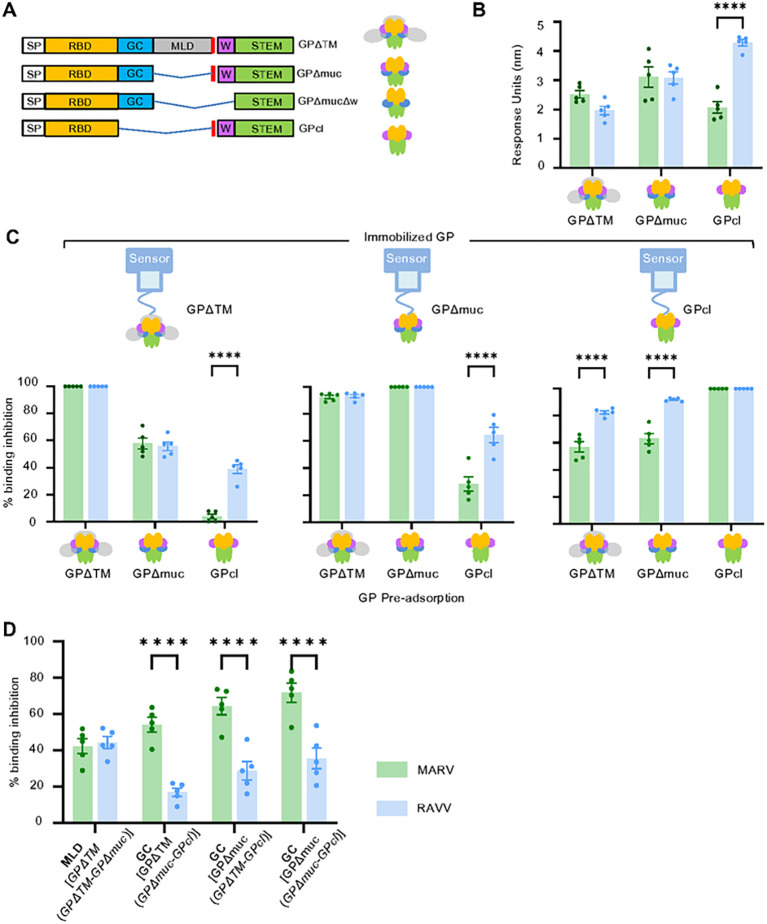
Proportion of binding-antibodies targeting GP regions. **(A)** Schematic of truncated GP forms. Blue lines represent deleted regions. SP, signal peptide; RBD, receptor binding domain; GC, glycan cap; MLD, mucin-like domain; W, wing; TM, transmembrane domain. Red line indicates the furin cleavage site. **(B)** Response Units (RU) of total serum binding to truncated GP forms. **(C)** Binding inhibition to immobilized GPΔTM, GPΔmuc or GPcI expressed as a percentage of total binding RU values obtained without serum pre-adsorption. **(D)** Proportion of the antibody response binding to the MLD and GC. The mean responses of sera are shown, with ± SEM. Significance between MARV and RAW vaccine recipients measured by two-way ANOVA with Sidak’s correction for multiple comparisons. ****P ≤ 0.0001.

We further assessed the level of homologous binding to truncated forms of GP (Fig. 3A); MARV or RAVV GP ectodomains (GPΔTM), mucin-deleted ectodomains (GPΔmuc, Δ257–425) and proteolytically-cleaved GPs (GPcl), and the wing-deleted RAVV GPΔmuc (GPΔmucΔw, additional deletion of residues Δ436–483). All GP forms were immobilized on Octet biolayer interferometry (BLI) sensors at comparable levels and allowed to bind antibodies in serum. In general, similar antibody binding levels were observed between MARV and RAVV vaccine-derived serum to immobilized GPΔTM or GPΔmuc from the respective virus (Fig. 3B). MARV-specific serum antibodies appeared to have a lower binding capacity to GPcl than RAVV-specific serum antibodies. Therefore, the response generated from MARV mRNA vaccination may target the glycan cap (GC) which is absent on the proteolytically cleaved form of GP more so than RAVV-vaccination.

### The MARV vaccine induces a greater antibody response to the GP glycan cap compared to the RAVV vaccine

The proportion of the vaccine-induced antibody response directed toward regions on MARV or RAVV GP [MLD, GC or receptor binding domain (RBD) of the GP1 subunit, and the wing and base regions of the GP2 subunit] was determined to identify the regions predominantly responsible for the binding antibody response to vaccination. GP region-specific responses were measured using BLI competition assays. Serum antibodies from vaccinated animals were allowed to bind a GP protein immobilized on the BLI sensor after pre-adsorption treatment with a GP variant (Fig. 3A) to remove antibodies targeting regions shared between the competing and immobilized GP. The proportion of MLD-specific antibodies was inferred from the percent of serum antibody binding to GPΔTM not removed by GPΔmuc pre-adsorption (Fig. 3C). MLD antibodies in the MARV vaccine recipient comprised approximately 40% of the response (Fig. 3D). The proportion of MLD antibodies in the RAVV vaccine recipients was similar to that of MARV recipients. GPcl, the protease cleaved form of GP, lacks the GC which is present in GPΔmuc and GPΔTM. We could therefore deduce the proportion of antibodies binding to the GC by subtracting the level of binding to GPΔTM removed by GPcl pre-adsorption from the level of binding to GPΔTM removed by GPΔmuc pre-adsorption (Fig. 3C,D). Approximately 55% of the MARV-directed response towards GPΔTM targeted the GC (Fig. 3D). However, the GC antibody proportion of the RAVV-directed response at ~ 17% was substantially lower than the MARV-directed response. When GPΔmuc was used as the capture ligand instead of GPΔTM (Fig. 3C), GC antibody proportions were augmented to 70% or 30% for the MARV or RAVV response (Fig. 3D), respectively, given the ratio of the surface areas of GC to GPΔmuc is greater than the ratio of GC to the full GPΔTM.

The fraction of the response towards the combined RBD, wing and GP2 regions was determined by the level of binding to GPΔTM or GPΔmuc inhibited by the presence of GPcl (Fig. 3C). The MARV-vaccine response towards this combined region was 30% less than the RAVV response. The greater proportion of GC antibodies relative to the total amount of binding antibodies in MARV recipients may have offset the response towards the combined RBD, wing and GP2 regions. However, the higher frequency of RAVV antibodies towards the combined RBD, wing and GP2 regions was confirmed using the reverse setting to calculate the level of binding to GPcl inhibited by GPΔmuc or GPΔTM pre-adsorption.

The proportion of the response directed to the wing domain could only be determined for RAVV due to the availability of RAVV-derived GPΔmucΔw. The level of binding to GPΔmuc following pre-adsorption of serum with GPΔmucΔw was used to calculate the frequency of the response towards the wing domain. Interestingly, GPΔmucΔw (436–483) had minimal effect on blocking antibody binding to all GP forms in the competition assays indicating that most of the response targeted the wing domain (Suppl. Figure 1A). However, in the reverse setting, serum antibody binding to immobilized GPΔmucΔw in the presence competing GP forms showed a substantial proportion of the response targeted shared regions, RBD and GP2 (Suppl. Figure 1B). Therefore, the actual wing domain antibody proportions in serum antibodies may be misrepresented in this assay system. The total response binding to GPΔmucΔw was poor (Suppl. Figure 1C). Furthermore, binding to the wing domain facilitates the structural rearrangement of GP to enhance binding of RBD antibodies (25). The absent wing domain on GPΔmucΔw may have prevented the sequestering of serum RBD antibodies by thwarting the cooperative recognition of RBD domains that occurs upon engagement of the wing domain.

Overall, these data demonstrate that both vaccines comparably target the MLD, but the MARV vaccine induces a greater antibody response to GC, while the RAVV vaccine induces a greater response towards the combined RBD, wing and GP2 regions.

### MARV and RAVV vaccines induce virus-neutralizing antibodies specific for different regions of GP

We next determined the regions on GP targeted by neutralizing antibody responses generated by the MARV or RAVV mRNA vaccine. Day 56 sera from MARV or RAVV-vaccinated guinea pigs were diluted to a concentration required to achieve at least 80% of neutralization. Diluted sera generated were then pre-absorbed with increasing concentrations of truncated GP proteins derived from the specific viruses targeted by each mRNA vaccine (MARV GPΔmuc and GPcl or RAVV GPΔmuc, GPΔmucΔw and GPcl). The presence of the truncated GP proteins sequestered antibodies that bound to regions shared with the full-length GP on the virus.

**Fig. 4.**
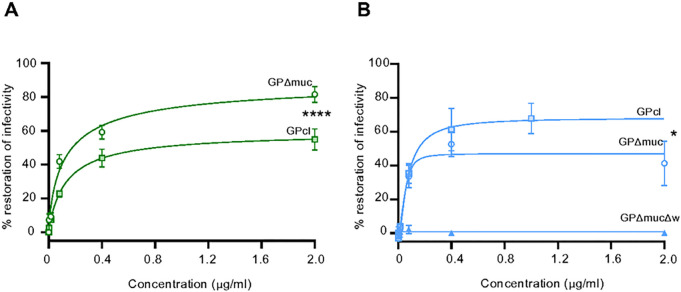
Epitope specificity of neutralizing antibodies. Neutralizing activity of day 54 **(A)** MARV or **(B)** RAW immune sera pre-absorbed with GPΔmuc and GPcI or GPΔmucΔw expressed as percentages of activity of the same sera not pre-absorbed with proteins. Sera from MARV or RAW vaccinated groups, diluted to achieve 80% of their neutralization activities, were incubated with increasing concentrations of truncated GPs from the relevant virus. Extra sum-of-squares F test was performed to determine significant differences in best-fit parameters between GPΔmuc and GPcI curves. *P ≤ 0.05 and ****P ≤ 0.0001.

The ability of serum from MARV mRNA vaccinated animals to neutralize the virus was nearly abolished with increasing concentration of MARV GPΔmuc, indicating that non-MLD-specific antibodies are major contributors to the neutralizing capacity of serum antibodies (Fig. 4A). The ability of non-MLD antibodies to neutralize virus in the presence of MARV GPcl was diminished to a lesser extent compared to neutralization in the presence of MARV GPΔmuc, indicating that the antibodies targeting the GC structure absent on MARV GPcl, contribute to virus neutralization.

For RAVV-vaccinated animals, non-MLD-binding antibodies also contributed to neutralization activity, although seemingly to a lesser extent than the MARV-derived antibodies since infectivity was not fully restored in the presence of RAVV GPΔmuc (Fig. 4B). While this finding may suggest that a substantial proportion of the RAVV-vaccine derived neutralizing antibodies targets the MLD, the presence of increasing concentrations of GPcl, the furin cleaved form of GP which lacks both the GC and MLD domains, restored virus infectivity to a better extent than did GPΔmuc. Accessibility to the RBD on the cleaved structure may enable improved sequestering of RBD-specific neutralizing antibodies. This finding also indicates that RAVV vaccine-derived neutralizing antibodies targeted the GP in its cleaved form better than the MARV-vaccine derived neutralizing antibody response.

Interestingly, RAVV virus infectivity was not restored in the presence of GPΔmucΔw, indicating that the RAVV wing domain is important for neutralization activity. Taken together, these data demonstrate that the GP regions targeted by neutralizing antibodies diverge between the two vaccines: the GC region was heavily involved in the neutralization response after MARV vaccination, while the RBD, wing and GP2 regions of the GPcl structure contributed substantially to the neutralization response following RAVV vaccination.

### The vaccines induce antibodies binding to protective epitopes in RBD and wing domain

**Fig. 5.**
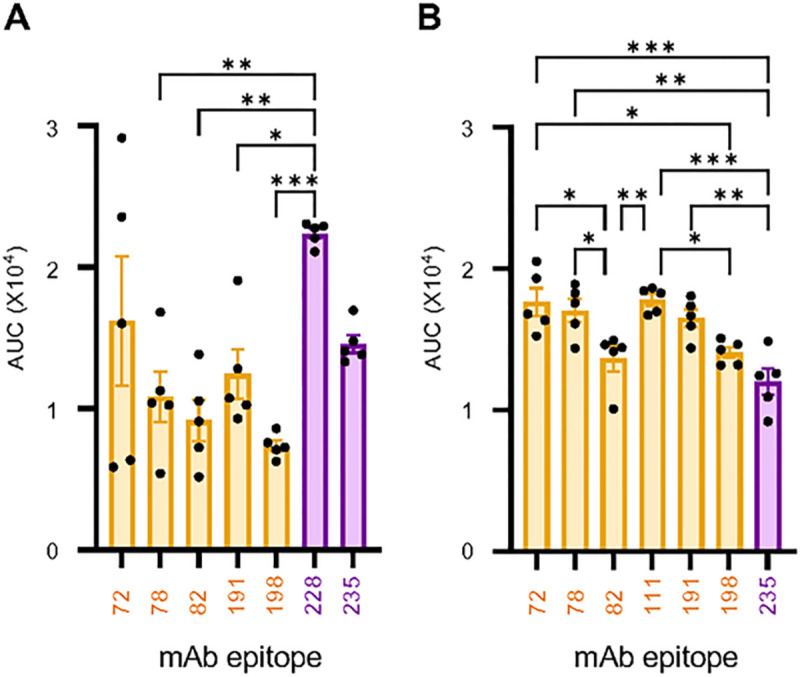
Prevalence of immune sera targeting known epitopes of human survivor mAbs. BLI-based competition-binding assay in which mAbs compete with day 54 pre-infection serum antibodies from **(A)** MARV or **(B)** RAW vaccine recipients for a specific epitope on biotinylated **(A)** MARV or **(B)** RAW GPs immobilized on streptavidin sensors. The level of inhibition is determined as a percentage of blocking activity compared to mock-control sera against the tested mAb. Symbols Indicate individual guinea pigs. Bars denote the average blocking of RBD mAbs (gold) or wing domain mAbs (purple) by sera from vaccinated groups ± SEM. Significance measured by one-way ANOVA with Tukey’s correction for multiple comparisons between the serum prevalence towards different mAb epitopes. *P ≤ 0.05, **P ≤ 0.01 and ***P ≤ 0.001.

We quantified the prevalence of the response directed to known protective epitopes in the wing and RBD domains of GP. Representative monoclonal antibodies (mAbs) isolated in our previous studies from human survivors with epitopes in the RBD (MR72, MR78, MR82, MR111, MR191 and MR198), and wing domain (MR228 and MR235) (25, 26) were selected to compete for binding to GP on the BLI platform with serum antibodies in samples collected after the booster dose (Suppl. Figure 2). MR111 was specific for RAVV GP, and all RBD-specific mAbs are neutralizing antibodies (26). The two mAbs specific to the wing domain do not neutralize virus but have Fc-mediated effector functions (25). MR228 only bound the wing domain of MARV GP, limiting the number of wing domain antibodies that could be used in competition for RAVV GP binding. Among these antibodies, MR72, MR78, MR82 and MR228 protected small animal models (25–27) while MR191 protected NHPs (27). Each of the representative RBD epitopes was targeted by a similar quantity of serum from recipients of the MARV mRNA vaccine (Fig. 5A). The response toward all RBD mAb epitopes, except for MR72, was lower in frequency than the response towards the wing domain epitope for MR228. The antibody frequency towards the wing domain epitope for MR235 was comparable with the RBD-targeted response. Conversely, the frequency of antibodies in RAVV mRNA recipients was similar towards most RBD epitopes, except the epitopes for MR82 and MR198. The frequency of antibodies targeting RBD epitopes was generally higher than the frequency directed towards the wing domain epitope for MR235 (Fig. 5B). Overall, the response recognition frequency to known epitopes in the RBD and wing domains appeared to differ between the MARV and RAVV mRNA vaccines.

### The vaccines induce antibodies specific for both cross-reactive and unique linear epitopes

The MARV and RAVV vaccine antibody response profiles were further scrutinized for any parallels or uniqueness in their linear epitope recognition. Linear epitopes of GP targeted by antibodies were characterized using peptide arrays designed with overlapping 15-mer peptides spanning the entire GP of MARV (strain Angola) or RAVV (strain Ravn), offset by 4 amino acids. Serum antibodies in samples collected after boost vaccination dose were allowed to bind each of the GP proteins to identify homologous or heterologous linear epitope recognition.

Linear epitopes located within the RBD (peptides 15 to 18, 23 to 30) and wing domains (peptides 109 to 116) of MARV and RAVV GP were recognized by both homologous and heterologous vaccine-induced antibodies. Moreover, the magnitude of vaccine-induced antibody binding to these epitopes within the homologous virus somewhat mirrored the magnitude of binding observed towards the same epitope in the heterologous virus. The RBD is highly conserved between the marburgviruses. Peptides 15 to 18, 23 to 25 and 29 to 30 encompass part of the engagement site for the host receptor Niemann-Pick C1 and the footprint for mAbs MR78 and MR191, which are neutralizing mAbs isolated from human survivors (28) that provide post-exposure protection to NHPs (23, 27). Peptides in the MARV GP2 wing domain recognized by both MARV and RAVV mRNA vaccine-specific antibodies encompass the epitopes for three mAbs, the two human mAbs MR235 and MR228 and the murine mAb 30G4 (25, 29). Peptides in the RAVV GP2 wing domain corresponding to the MR228 epitope were not recognized by MARV and RAVV mRNA vaccine-specific antibodies (peptide 112). MR228 failed to bind RAVV GP due to a two amino acid difference (aa 454T-455E) in the epitope compared to MARV GP (aa 454A-455P). This amino acid divergence appears to diminish the recognition potential of the humoral response towards RAVV.

Antibodies targeted to linear epitopes in the MLD were unique to the respective viruses, with MARV-MLD antibodies unable to recognize the RAVV-MLD and vice versa. RAVV-vaccine antibodies had a greater breadth of binding to the MLD than MARV-derived antibodies. MLD is a poorly conserved region between the two viruses, and therefore the lack of recognition of MLD in heterologous viruses was not unexpected.

Interestingly, MARV mRNA vaccination induced antibodies with a greater capacity to bind the internal fusion loop (IFL) region of both MARV and RAVV, compared to the RAVV mRNA-vaccination. Weak recognition of the IFL by RAVV-vaccine antibodies indicates they have lower affinity than MARV vaccine antibodies, the RAVV IFL is poorly ranked amongst the immunogenic B-cell epitope hierarchy or the RAVV GP2 stem is somewhat obstructed (29). MARV mRNA vaccination also induced antibodies with a greater capacity to bind the GP2 stem region of both MARV and RAVV GP compared to RAVV mRNA vaccination. Antibody recognition of the stem was generally weak, with detection of peptide 149 in the heptad repeat-1 (HR1) of both MARV and RAVV GPs being strongest in the region. While protective mAbs targeting the stem have not been identified for marburgviruses thus far, an indication of rarity, mAbs specific for the GP stem of ebolaviruses, have been isolated from human survivors (30).

The peptide arrays highlight regions within GP that are virus-specific and regions that are cross-reactive. While the homologous antibody response following vaccination was greater in breadth and magnitude toward the linear epitopes from the respective virus, a comparable magnitude of recognition was observed for heterologous binding at cross-reactive epitopes. The strongest binding was observed for the RBD of GP1 and the wing of GP2. The linear epitopes recognized by cross-reactive vaccine-induced antibody populations may be important contributors to their ability to cross-neutralize the two viruses.

### The vaccines induce multiple Fc mediated effector functions

**Fig 7.**
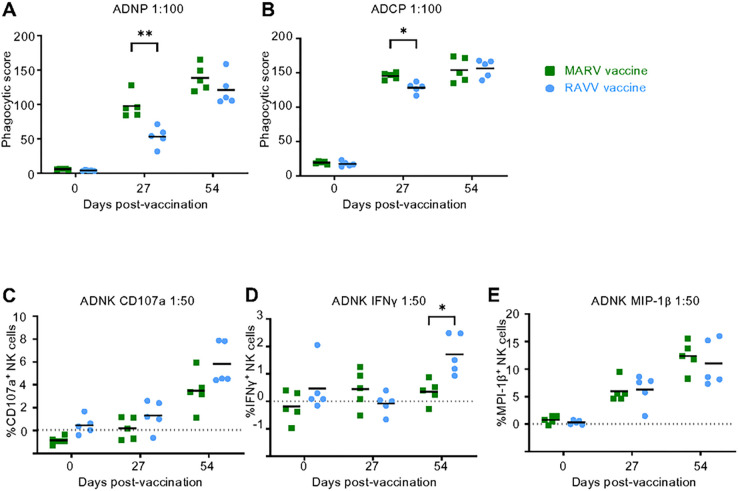
Fc-mediated antibody responses. The level of **(A)** ADNP, **(B)** ADCP and **(C-E)** ADNK activity of immune sera at specified dilution, in guinea pigs grouped according to vaccine at days 27 or 54 post-vaccination. NK cell activation measured according to production of **(C)** CD107a, **(D)** IFNγ and **(E)** MIP-1β. Significance was measured by repeated measures two-way ANOVA with Sidak’s correction for multiple comparisons between vaccine groups. *P ≤ 0.05 and **P ≤0.01.

In addition to mechanical neutralization by antibodies, their Fc-mediated effector functions have been implicated in contributing to protection in vaccinated and natural infection survivors (31). We examined the ability of the MARV and RAVV vaccine-induced immune sera to activate phagocytosis mediated by neutrophils (antibody-dependent neutrophil phagocytosis; ADNP) and monocytes (antibody-dependent cellular phagocytosis; ADCP). Antibody responses produced after MARV or RAVV mRNA vaccination activated virus strain-specific ADNP and ADCP functions *in vitro*. ADNP and ADCP activities were higher after prime MARV vaccination compared to after RAVV vaccination, but activities increased after the boost dose such that discernable differences were not observed between the two vaccines (Fig. 7A,B).

We also examined the ability of serum antibodies to facilitate antibody-dependent natural killer (ADNK) cellular cytotoxicity by measuring their markers for degranulation (CD107a) and activation (macrophage inflammatory protein-1β [MIP-1β] and interferon-γ [IFNγ]). Activation of the NK cellular activity was achieved by both vaccines (Fig. 7C–E). The MARV and RAVV boosters were required to activate similar levels of degranulation. The levels of NK cells positive for MIP-1β were comparably increased after prime vaccinations with both vaccines and further elevated by the booster vaccinations. Almost no NK cells positive for IFNγ were detected after prime vaccination; unexpectedly, the levels increased after a booster of the RAVV but not the MARV vaccine. NK cells may control infection directly by their cytolytic functions and only partially by relying on their recruitment of other immune cells through MIP-1β, but not IFNγ production.

### mRNA vaccines protect against MARV and RAVV infections

**Fig 8.**
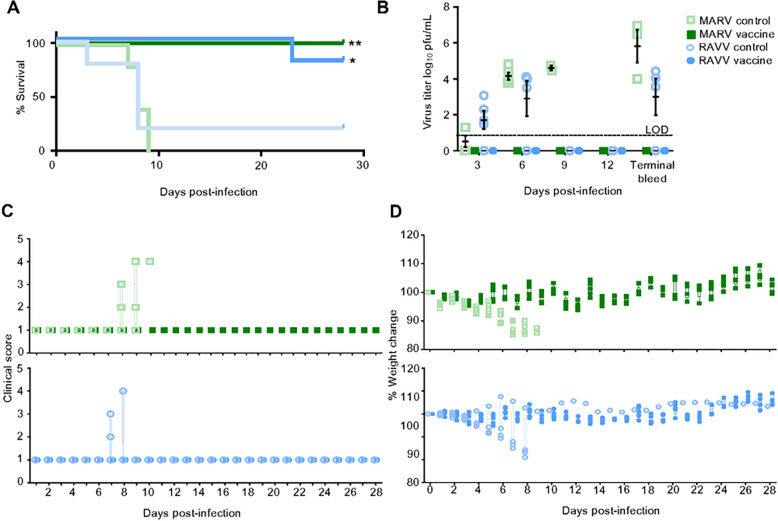
mRNA vaccine efficacy against homologous virus infection in guinea pigs. Guinea pig recipients of the MARV mRNA (dark green), RAW mRNA (dark blue) or mock vaccines (light blue and green) were challenged with 1,000 PFU of the corresponding guinea pig-adapted MARV or FLAW by the IP route. All animals were monitored for changes in **(A)** survival, **(B)** viremia, **(C)** clinical scores, and **(D)** % weight change over 28 days. **(B)** Serum was collected from all animals at 3-day Intervals over the course of infection and measured for infectious virus determined by plaque titration (PFU/mL). Limit of detection (LOD). Mean values ± SEM. **(A)** One RAW vaccinated guinea pig was euthanized on day 23, due to a sustained physical injury. This animal maintained a steady weight and had no detectable virus during the infection phase and at the time of euthanasia. Mantel-Cox test for comparison of survival curves from mock and vaccine groups infected with MARV or FLAW (*P ≤ 0.05 and **P ≤ 0.01).

At day 56, guinea pigs were challenged with 1,000 plaque-forming units (PFU) of guinea pig-adapted MARV strain Angola (32) or guinea pig-adapted RAVV strain RAVV (33), respectively (Fig. 1). All guinea pigs vaccinated against MARV or RAVV survived infection (Fig. 8A). Over the 28-day infection phase, serum was collected at 3-day intervals for the first 12 days to measure for viremia (Fig. 1). Guinea pigs were also monitored for clinical signs of disease including lethargy, neurologic signs and weight loss. Vaccinated guinea pigs maintained steady weight over the infection phase (Fig. 8D), did not have detectable viremia (Fig. 8B), and displayed no signs of disease (Fig. 8C). One RAVV-vaccinated guinea pig sustained a physical injury unrelated to infection and was euthanized at day 23 (Fig. 8A). No virus was detected in the blood of this animal collected at the time of euthanasia. Control RAVV-infected guinea pigs developed severe disease and exhibited weight loss before succumbing to infection by day 9. Four out of 5 MARV-infected control guinea pigs succumbed by day 8, and exhibited clinical disease and weight loss over the infection course (Fig. 8C,D). The shorter time to lethality with MARV infection compared to RAVV is consistent with its greater virulence observed in the guinea pig model (33). The lack of detectable circulating virus in the surviving RAVV control guinea pig (Fig. 8B) may indicate imprecise administration of the infectious inoculum by intraperitoneal injection, a strict requirement to achieve uniform lethality.

## DISCUSSION

The mRNA vaccines against MARV and RAVV we developed here successfully protected guinea pigs against death and severe disease caused by lethal challenge with the respective viruses. mRNA vaccines proved to be highly efficacious in preventing severe disease caused by SARS-CoV-2 infection. However, mRNA-LNP vaccine-induced immunity against SARS-CoV-2 is non-sterilizing in humans and NHPs at its clinically-relevant dose (34) with limited durability that has enabled continued virus transmission and breakthrough infections by evolving virus variants of concern. An understandable level of uncertainty may surround the ability of mRNA-LNP vaccines to protect against a highly lethal pathogen, where a robust near-sterilizing immune response is crucial in preventing disease progression. While filoviruses do not mutate as fast as coronaviruses in the field during outbreaks, the importance of curbing virus replication becomes increasingly important as filoviruses are highly transmissible. In our study, the immunity conferred by mRNA-LNPs vaccination was potentially sterilizing as both marburgviruses remained undetectable in circulation. The mRNA-encoded marburgvirus GP antigen is likely to be sufficiently immunogenic to achieve sterilizing immunity.

We sought to characterize the antibody responses to both vaccines to determine similarities and differences in the profiles given the sequence diferences between MARV and RAVV GPs. While mRNA vaccine sequences are easily tailored to target the pathogen of concern, a vaccine that can generate a broadly-specific response that covering more cross-protective antigens is highly desirable. The frequency of the antibody response towards certain GP domains differed between the two vaccines. MARV mRNA vaccine generated a greater frequency of antibodies that targeted the GC than the RAVV mRNA vaccine. Moreover, recognition of homologous peptides in the GC was more evident with the MARV response, indicating that this region in MARV strains may be more immunogenic than in RAVV. The proportion of the total antibody response towards the MLD was similar between the RAVV and MARV vaccines. However, the breadth of recognition towards autologous linear MLD epitopes was greater for RAVV vaccine recipients suggesting the sequence heterogeneity at the MLD shaped the epitope-recognition profile without affecting the binding frequency.

Differences between MARV and RAVV vaccine-derived neutralizing antibody responses were also identified. RAVV mRNA vaccination appeared to generate more neutralizing antibodies towards the furin cleaved form of GP (GPcl), rather than the un-cleaved structure of GP lacking the MLD (GP∆muc), a finding that may be attributed to the RBD epitopes exposed following the removal of the GC structure. On the other hand, the MARV mRNA vaccine appeared to generate a greater neutralizing response towards GP∆muc than GPcl. Therefore, GC epitopes appear to be involved in neutralizing activity of MARV; GC antibodies in serum that were not removed by pre-adsorption with GPcl (which lacks the GC) could neutralize the virus. The strength of linear epitopes recognition in the GC of MARV, but not RAVV recipients, indicates that it is a virus-specific immunogenic region. It was previously suggested that mAbs recognizing the regions in proteolytically cleaved GP, in addition to the RBD, were involved in virus neutralization (25). Neutralizing mAbs from MARV survivors are rare and diminish over time (35, 36). While no GC-specific mAbs from survivors have been described in the literature thus far, the possibility of their existence cannot be ruled out given our findings of linear epitopes footprints and a large portion of the vaccine-derived antibody composition associated with the region. The GC of MARV GP appears to shape the serum antibody response profile more so than the GC of RAVV GP. GC elicited higher antibody frequencies, neutralizing capacity and linear epitope recognition over RAVV. Conversely, RAVV RBD, wing and GP2 domains combined, generated proportionally greater antibody binding and neutralizing responses compared to MARV. Unlike ebolaviruses, the GC structure of marburgviruses appears disordered, such that the RBD domain is exposed even prior to cathepsin cleavage (23). The GP of MARV and RAVV are thought to have similar structures. However, RAVV GP structure is more stable compared to MARV GP (29). Our results suggest that sequence evolution may influence a structural divergence between the GPs of distant marburgviruses by potentially affecting stability, modifications (37) and/or the spatial location of domains. The crystal structures of GP from closely related ebolaviruses, EBOV and SUDV, highlighted electrostatic differences which may be responsible for their opposing susceptibility to endosomal proteases (38). In this study, the intact GP of MARV appeared to be more immunogenic over its proteolytically-cleaved structure, promoting a GC-heavy response. The proteolytically cleaved form of RAVV appeared more immunogenic than its full structure. A structural divergence may also explain the varied responses towards the RBD, IFL or the GP2 stalk of MARV and RAVV despite these sites sharing near 100% sequence homology.

Previously, protective mAbs for survivors targeting the wing domain were shown to possess Fc-effector functions. In another study, the wing domain elicited protective antibodies in mice with partial *in vitro* neutralizing activity (29). We show that the wing domain may be heavily involved as a target for recognition by neutralizing serum antibodies. Removal of RBD, GC and GP2 antibodies in sera from RAVV vaccinated animals by pre-absorption with GPΔmucΔw did not eliminate virus neutralization. Therefore, antibodies can directly target the wing domain for neutralization and not just effect a conformational change in GP that enables access by RBD neutralizing antibodies (25). The MLD domain of GP encoded by both the MARV and RAVV mRNA vaccines did not appear to be heavily involved in the neutralizing potential of the humoral response.

The linear epitope footprint encompassing MR228 and MR235 in the GP2 wing region was a prominent site recognized by MARV and RAVV vaccine-induced serum antibodies. Despite MR228 and MR235 having overlapping epitopes, the MARV mRNA vaccine-directed response towards MR228 exceeded that of MR235. The frequency of antibodies in MARV-vaccinated recipients targeting the MR228 epitope was greater than that of antibodies targeting RBD epitopes, while the frequency of antibodies targeting the MR235 epitope were similar to RBD antibodies. This finding conflicts with previous findings in which the prevalence of wing domain antibodies in the serum of human MARV survivors was lower compared to RBD antibodies (25). However, for RAVV mRNA vaccine recipients, the frequency of most RBD-specific antibodies was generally higher than MR235. MR228 is a non-neutralizing mAb that uses Fc-effector functions to protect animals from lethal infection, potentially highlighting the importance of its epitope in directing cell-mediated immune responses (39). The varying response towards RBD and wing domain mAb epitopes generated by the two vaccines points to an epitope-driven disparity influencing the functional antibody profile.

Antibody-dependent cellular functions contribute to protection against MARV infection in the absence of detectable neutralizing activity (40). Both MARV- and RAVV-specific serum antibodies facilitated ADCC activity to a similar extent after booster doses. However, the antibodies with phagocytosis potential were induced faster in MARV recipients compared to RAVV recipients, despite both vaccines eliciting similar IgG kinetics (Fig. 3). The superior activity of antibody-mediated cellular phagocytosis in MARV-recipients and neutralization in RAVV-recipients suggest that differences in the GP regions targeted by the two vaccines may influence antibody functionality and protective mechanisms. The apparent greater phagocytosis potential of MARV vaccine-derived antibodies may be attributed to a somewhat skewed recognition intensity toward linear epitopes and protective mAb epitopes in the wing domain compared to the RBD. The wing domain is part of GP2, equatorially projected on GP. It is thought to be recognized primarily by antibodies with Fc-mediated effector functions given the spatial accessibility to immune cells (25). The epitopes of human survivor mAbs with ADCC functions were targeted by antibodies in serum from our mRNA-vaccinated guinea pigs. Fc effector functions likely contribute to the protective mechanism of the vaccines, but their contribution and the exact protective mechanisms may differ between the two vaccines.

The ability to induce broad protection against both marburgviruses appears to depend on the vaccine platform. A Venezuelan equine encephalitis virus replicon vaccine against MARV (strain Musoke) failed to protect against RAVV (41, 42). Conversely, a VSV-vectored MARV vaccine (strain Musoke) did confer protection against RAVV (43). While we did not directly test the cross-protective effects for each of our mRNA-LNP compositions against heterologous virus, we showed cross-neutralization antibody responses were induced against MARV and RAVV, with the RAVV mRNA vaccine promoting somewhat better cross-neutralization. Peptide array analysis identified common linear peptides within two regions of GP, the RBD and wing, that were recognized by heterologous-vaccine induced antibodies. Therefore, the mRNA vaccine has the potential to induce broadly reactive responses, but confirmation of this capacity will be in the form of future challenge studies with heterologous viruses. Nonetheless, the nature of the mRNA platform enables combinatorial sequence, cocktail, or multi-dosing approaches against several viruses, without risk of generating immunity towards the actual platform. A cross-protective filovirus mRNA vaccine, using a sequence either optimized or representing a mixture of two mRNAs, is likely achievable.

The differences in antibody reactivity and functionality profiles between the two marburgvirus vaccines we identified in this study are equally important as they are insights into potential structural differences in the targeted GP antigen. This finding may have fundamental implications in designing a vaccine that is cross-protective against both marburgviruses. These differences potentially influence responses towards regions with sequence homology between strains, which under conventional circumstances, would elicit similar immune responses. Our promising results against EBOV previously and now against MARV and RAVV support future preclinical efficacy testing of the mRNA-LNP platform in the stringent NHP model.

## METHODS

### mRNA synthesis and nanoparticle formulation

mRNA vaccines were synthesized *in vitro* by T7 polymerase-mediated transcription with substituted 1-methylpseudo UTPs, using linearized DNA templated encoding GPs from MARV isolate Angola05, GenBank accession number: DQ447653.1 and RAVV (isolate Kenya 1987, GenBank accession number: DQ447649.1). The wild-type signal sequence of GP and 5′ and 3′ untranslated regions (UTRs) (24) were incorporated into the mRNAs. The mRNAs were purified and resuspended in a citrate buffer at the desired concentration. A donor methyl group S-adenosylmethionine (SAM) was added to methylated capped RNA (cap-0), resulting in a cap-1 structure to increase mRNA translation efficiency (44). LNP formulations were prepared as previously described (24).

### Testing of the immunogenicity and protective efficacy in guinea pigs

Two groups of adult female guinea pigs, strain Hartley (n = 5), were intramuscularly vaccinated in the left hind leg on days 0 and 27 with 0.1 mL of MARV and RAVV mRNA vaccines (40 μg). Two groups were mock-vaccinated with phosphate buffered saline (PBS) to serve as the MARV or RAVV infection controls. On day 56, the mock-vaccinated and vaccinated groups were intraperitoneally infected with 1,000 PFU of the respective guinea pig-adapted MARV (provided by Dr. G. Kobinger while at the National Microbiology Laboratory, Winnipeg, Canada) or RAVV (33). Guinea pig-adapted MARV was originally isolated from a patient in Angola, passaged once in Vero-E6 cells, eight times in Hartley guinea pigs using liver and spleen homogenates, once in Vero PP cells, and once in Vero cells for stock production. Guinea pig-adapted RAVV was developed by 2 passages in strain 13 guinea pigs and 1 passage in Hartley guinea pigs. Serum was collected days 0, 27, and 54 post-vaccination and at 3-day intervals over 12 post-infection days, and at day 28 post-infection, the time of euthanasia. The animal experiment was approved by the University of Texas Medical Branch Institutional Animal Care and Use Committee.

### Analysis of viremia

Vero-E6 cells were inoculated with 10-fold serially diluted serum in MEM (Thermo Fisher Scientific) containing 0.05 mg/mL gentamicin (Thermo Fisher Scientific). After 1 hour absorption at 37°C, the inoculum was replaced with carboxymethyl cellulose overlay, plates were incubated for 4 days, monolayers were fixed with formalin, and plaques were immunostained.

### Plaque reduction assays

Plaque reduction neutralization assays were performed as previously described (17). Viruses used for neutralization-based assays were MARV strain 200501379 Angola isolated during the outbreak in Angola in 2005 (45) and passaged three times in Vero-E6 cells. MR186 and MR198 human mAbs, known to possess neutralizing activity for MARV or RAVV respectively, starting at 200 μg/μL were included as positive controls for the assay. After 4 days, plates were fixed and immunostained. Plaques were counted at the antibody titer at which 60% neutralization was achieved was calculated.

### Enzyme linked immunosorbent assays

Enzyme linked immunosorbent assays (ELISAs) were performed as described (17) with modifications. Briefly, 96-well high binding microlon plates (Greiner) were coated overnight at room temperature with MARV (strain Angola) or RAVV (strain Ravn) GP (8 ng/well, IBT Bioservices). Plates were blocked with 3% milk powder in phosphate buffered saline (PBS). Sera diluted 4-fold in blocking solution starting at 1:16 was added to the plate. A peroxidase-labeled goat anti-guinea pig IgG (1:5,000 dilution; Jackson ImmunoResearch Laboratories) detected bound antibodies. Blocking and binding steps with sera or secondary antibodies were performed at 37°C for 1 h.

### Serum binding and competition assays

A FortéBio Octet Red96 instrument (Sartorius) was used to measure serum antibody binding to MARV or RAVV GPs and their intermediate forms. All assays were performed with agitation at 1,000 rpm, at 28°C in black 96-well plates. All samples were diluted in 1× Kinetics buffer (FortéBio) with a final volume of 200 μL per well. Biotinylated GP, GPΔmuc (Δ257–425), GPΔmucΔw (additional residues Δ436–483) or GPcl were immobilized onto streptavidin sensors for 300 s to capture ~ 1 nm, with variability within a row of sensors not exceeding 0.1 nm. Biosensor tips were then equilibrated for 300 s in 1× Kinetics buffer before binding measurements. Sera were diluted 1:50, and binding was assessed for 600 s, followed by dissociation for 600 s in 1× Kinetics buffer. Parallel corrections for baseline drift were made by subtracting measurements recorded with GP-loaded sensors in the absence of sera.

For pre-adsorption studies, sensors were treated with biocytin for 120 s after immobilization of a biotinylated GP form. Sera depleted with excess amounts of GP forms of MARV (5 μg GPΔTM, GPΔmuc, and 2.5 μg GPcl) or GP forms of RAVV (7.5 μg GPΔTM and GPΔmuc, 5 μg GPΔmucΔw and 1 μg GPcl) were allowed to bind to sensors as described above. To determine nonspecific binding responses, binding of sera from mock-vaccinated animals to GP variant-loaded probes was monitored and set as the background. We calculated the percent inhibition of binding to an immobilized GP after serum adsorption relative to the binding observed without pre-adsorption using the following formula: % inhibition = 100 – {[binding of serum pre-adsorbed with GP form (nm)/binding of serum without pre-adsorption (nm)] × 100}. The percent inhibition values, derived from one immobilized GP variant as the common denominator, were used to calculate the relative proportions of serum binding to a specific GP domain.

For site-specific antigenicity assessment, GP-loaded sensors (captured at ~ 0.5 nm) were incubated with serially diluted serum in 1× Kinetics buffer for 900 s to saturating signal against the competing mAb. Probes were then washed for 120 s before the reactivity of competing mAbs specific for the RBD (MR72, MR78, MR82, MR111, MR191 and MR198) or wing domain (MR228 and MR235) was assessed for 600 s. All mAbs recognize both MARV and RAVV, except for MR111 and MR228 which were specific for RAVV or MARV, respectively. GPΔmuc-loaded sensors were used for competition with all mAbs except MR235 which only bound GPΔTM. The binding inhibition to GP was calculated as a percentage of the blocking activity of sera from vaccinated animals compared to the mock-vaccinated control sera against the tested mAb. Data analysis and curve fitting were carried out using Octet software, version 7.0.

### Reversing neutralizing activity in the presence of GP forms

Competition neutralization assays were performed as previously described (46). Briefly, day 54 sera diluted to concentrations that neutralized at least 70% of MARV or RAVV were incubated in duplicate with increasing concentrations of MARV or RAVV GPΔmuc or GPcl. GPΔmucΔw was also included for RAVV-specific serum. Preabsorbed serum was then exposed to virus in a neutralization assay. The ability of GP forms to reverse the neutralizing activity of serum (restoration of virus infectivity) was calculated as a percentage of the plaques formed in the presence of serum incubated with the competing GP forms compared to the serum without the GP forms.

### Binding of the immune sera to peptide microarrays

Peptide microarrays were used to map the linear epitopes in MARV and RAVV GP recognized by the humoral response to vaccination. A microarray slide consisted of 21 blocks to enable analysis of up to 20 samples and one secondary antibody control. Each block was spotted with 168, 15-meric peptides offset by 4 amino acids, spanning the 681 amino acids of GP of MARV strain Angola (UniProtKB/Swiss-Prot: Q1PD50.1) or RAVV strain Ravn (NCBI Reference sequence: YP_009055225.1), as triplicate subarrays, by JPT Peptide Technologies GmbH (J.P.T.). Serum diluted 1:200 in wash buffer (J.P.T.) was applied to individual chambers on the slides and incubated for 1 h at 30°C. Following 4 washes, slides were incubated with 0.1 μg/mL anti-guinea pig IgG Cy5-conjugated antibodies (Jackson ImmunoResearch Laboratories). After additional washes and a final rinse in deionized water, the slide was dried by centrifugation. Slides were scanned with the GenePix 4200AL using the 635 nm laser at 500 PMT and 100 Power settings. The fluorescent intensities for each spot of the array image were analyzed by GenePix Pro 6 (Molecular Devices), and the MFI across the triplicate sub-arrays for each block was calculated and normalized by subtraction from the secondary antibody control. Sera from all animals per group were tested, and normalized MFIs for each peptide were corrected for baseline by subtracting the corresponding pre-vaccination MFIs.

### Fc-medicated effector functions

Antibody-dependent NK cell degranulation: Recombinant MARV or RAVV GP (IBT Bioservices) was coated onto MaxiSorp 96-well plates (Nunc) at 300 ng/well at 4°C for 18 h. The wells were washed three times with PBS and blocked with 5% bovine serum albumin in PBS. Sera from immunized guinea pigs diluted 1:50 in PBS were added, and the plates were incubated for 2 h at 37°C. Unbound antibodies were removed by washing three times with PBS, and human NK cells freshly isolated from the human donor peripheral blood by negative selection (Stem Cell Technologies) were added at 5 × 10^4^ cells/well in the presence of 4 μg/mL brefeldin A (Sigma-Aldrich), 5 μg/mL GolgiStop protein transport inhibitor (Life Technologies, Carlsbad, CA), and anti-CD107a antibody (1:40 phycoerythrin [PE]-Cy5; clone H4A3; BD Biosciences). The plates were incubated for 5 h. Cells were stained with anti-CD3 (1:100 Alexa Fluor 700; clone UCHT1; BD Biosciences), anti-CD16 (1:100 allophycocyanin [APC]-Cy7; clone 3G8; BD Biosciences), and anti-CD56 (1:100 PE-Cy7; clone B159; BD Biosciences), followed by fixation and permeabilization with the Fix & Perm reagent (Life Technologies) according to the manufacturer’s instructions to stain for intracellular IFN-γ (1:50 APC; clone B27; BD Biosciences) and MIP-1β (1:50 PE; clone D21–1351; BD Biosciences).

Antibody-mediated neutrophil (ADNP) or cellular (monocyte, ADCP) phagocytosis: Recombinant MARV GP or RAVV GP were biotinylated using Sulfo-NHS-LC-LC biotin (Thermo Fisher Scientific) and coupled to 1-μm FITC + NeutrAvidin beads (Life Technologies). Sera from vaccinated guinea pigs were diluted 1:100 in cell culture medium and incubated with GP-coated beads for 2 h at 37°C. Neutrophils isolated from donor peripheral blood, were added at a concentration of 5.0 × 10^4^ cells/well, and the plates were incubated for 1 h at 37°C. The cells were stained at 1:100 with CD66b (Pacific Blue; clone G10F5; BioLegend), CD3 (Alexa Fluor 700; clone UCHT1; BD Biosciences), and CD14 (APC-Cy7; clone MφP9; BD Biosciences). Neutrophils were defined as positive for a high side scatter area (SSC-Ahigh), CD66b+, CD3−, and CD14−. ADCP was measured as previously described (47) using a human monocyte cell line (THP-1 cells). Briefly, THP-1 cells (2.0 × 10^4^ cells per well) were for 18 h at 37°C with the GP-coated FITC bead serum mixtures in duplicate. All cells were fixed with 4% paraformaldehyde.

Stained cells from ADNK, ADNP and ADCP assays were analyzed by flow cytometry on a BD LSRII flow cytometer, and a minimum of 30,000 (ADNP) or 10,000 (ADCP) events were recorded and analyzed. The phagocytic score was determined using the following formula: [(percentage of FITC + cells) × (median fluorescent intensity [MFI] of the FITC + cells)]/10,000.

### Statistics

Statistical tests to determine the P values were calculated using GraphPad software, Inc. and are indicated in the figure legends. Differences were considered significant when P < 0.05.

## Figures and Tables

**Fig. 6.**
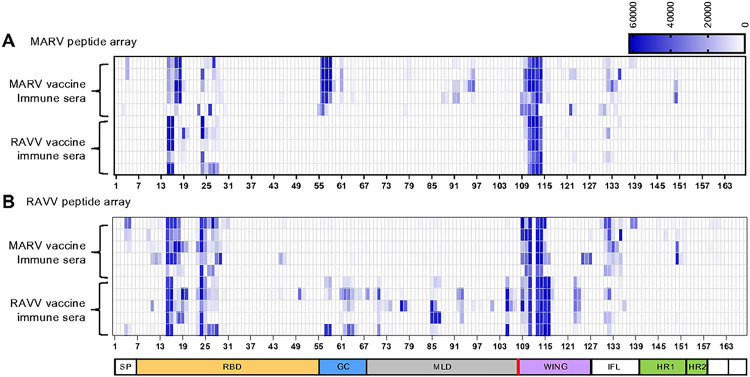
Binding of the immune sera to peptides spanning full-length MARV or RAW GP. The mean fluorescence intensity (MFI) of day 54 immune sera from each guinea pig binding individual peptides spanning RAW **(A)** or MARV **(B)** GP plotted against each peptide (x-axis) in a heat map. The domain schematic of GP identifies the location of each peptide within GP. SP, signal peptide; RBD, receptor binding domain; GC, glycan cap; MLD, mucin-like domain; W, wing; IFL, internal fusion loop; HR1, heptad repeat 1; HR2, Heptad repeat 2. Red line denotes the furin cleavage site. The vaccine constructs are indicated at the left y-axis of each heat map. Bars represent the average MFI.
